# A case of central retinal artery occlusion following embolization procedure for juvenile nasopharyngeal angiofibroma

**DOI:** 10.4103/0301-4738.67065

**Published:** 2010

**Authors:** Alireza Ramezani, Hamidreza Haghighatkhah, Habibollah Moghadasi, Morteza S Taheri, Hiva Parsafar

**Affiliations:** Department of Ophthalmology, Imam Hossein Medical Center, Shaheed Beheshti Medical University, Tehran, Iran; 1 Department of Radiology, Shohada-e-tajrish Medical Center, Shaheed Beheshti Medical University, Tehran, Iran; 2 Department of Otolaryngology, Loghman-e-hakim Medical Center, Shaheed Beheshti Medical University, Tehran, Iran

**Keywords:** Central retinal artery occlusion, embolization, juvenile nasopharyngeal angiofibroma

## Abstract

A 23-year-old male patient with right nasal Juvenile Nasopharyngeal Angiofibroma (JNA) developed Central Retinal Artery Occlusion (CRAO) during embolization of the tumor using polyvinyl alcohol particles before endoscopic excision. Classic CRAO management was initiated by an ophthalmologist after 12 h. Retrospective evaluation of the angiograms revealed a tiny communication between the external carotid and ophthalmic arteries which had not been noticed before embolization. During endoscopic excision, the tumor was found to originate extraordinarily from midline structures. It was concluded that CRAO might be a rare complication of JNA embolization. Careful preoperative angiographic evaluations to detect communicating arteries and immediate ophthalmologic consultation in case of developing visual symptoms during the procedure are necessary.

Central Retinal Artery Occlusion (CRAO) is one of the most sudden and dramatic events seen by ophthalmologists. One of the major causes of CRAO is embolism. There are many procedures associated with embolic complications and interventional radiological procedures are among the rare ones.[1]

Juvenile Nasopharyngeal Angiofibroma (JNA), one of the common benign nasal cavity tumors of adolescence, exhibits a strong tendency to bleed during surgical removal. Nowadays, preoperative embolization is commonly used to minimize such intraoperative bleeding; however, this technique is not without compli-cations.[2,3] Here, we present a rare case of CRAO which occurred following preoperative embolization in a patient with JNA. It also provides an opportunity to discuss two pitfalls which occurred during the patient’s management.

## Case Report

A 23-year-old male patient presented with the com-plaint of right nasal obstruction. After observing a purple pink mass completely filling the right nasal cavity, the diagnosis of JNA was made. Computerized tomography revealed a nasal cavity mass extending partially to the sphenoid sinus, pterygomaxillary fossa, and infratemporal fossa.

**Figure 1:**
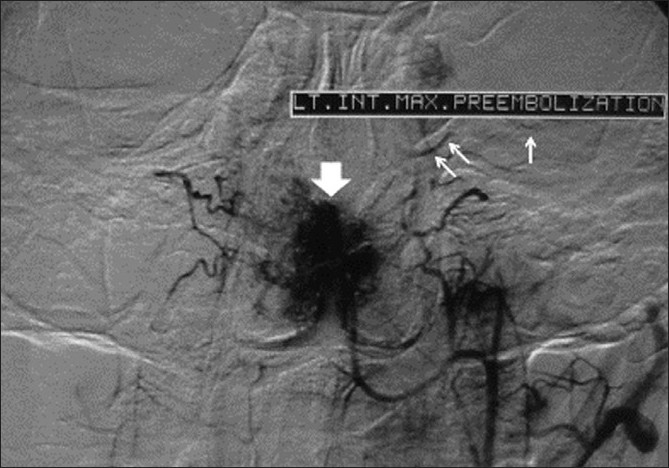
Pre-embolization angiogram demonstrating tumor bush (arrow head) and a suspicious tiny communicating artery between external carotid and ophthalmic arteries (arrows)

In order to reduce bleeding during the later endoscopic resection, the patient underwent a transarterial particulate embolization procedure as a part of preoperative preparation. After catheterization of both external carotid arteries with 5 F MP catheters (Cordis) via right transfemoral approach, selective angiography was performed. Abnormal tumor blush [Fig. 1, arrowhead] was noted with bilateral supply from both internal maxillary and ascending pharyngeal arteries. Neither extracranial-intracranial communication nor external-internal carotid collateral artery was detected. Therefore, embolization process was carried out bilaterally using Polyvinyl Alcohol (PVA) particles (150-250 micrometer) through both internal maxillary and ascending pharyngeal arteries. The progress of the vascular occlusion during embolization was monitored with repeated hand injection of contrast mediath. Before finishing the embolization the patient complained of sudden loss of vision in his left eye. After a control angiography, the procedure was stopped and 5000 IU heparin was injected intravenously. Immediate post-embolization angiograms demonstrated a successful reduction of the tumor blush with no reperfusion.

After 12 h, the patient was examined by an ophthalmologist in another hospital. Left eye visual acuity (VA) was counting finger at 0.5 meter and the relative afferent pupillary defect was positive in this eye. Left fundus examination revealed retinal edema and a ″cherry-red spot″ appearance of the macula with narrowed vessels, which were compatible with the diagnosis of CRAO. Ocular massage, anterior chamber paracentesis as well as systemic therapy with carbonic anhydrase inhibitor and mannitol were initiated. During the follow-up, VA stabilized at 20/200.

Retrospective and precise reevaluation of pre-embolization angiograms revealed a suspicious tiny communication between the external carotid artery and ophthalmic vessels on the left side [Fig. 1, arrow]. Such a communication was not found on the right side.

Four months after the embolization process, the patient underwent a successful endoscopic angiofibroma en bloc exci-sion. Interestingly, the tumor was found to originate from midline structures and attached to the posterior free border of the nasal septum.

Fourteen months after embolization, VA was 20/200. Cherry-red spot disappeared leaving attenuated retinal arterioles and optic disc atrophy.

## Discussion

Many CRAOs are embolic in origin. Radiologic procedures including hysterosalpingography, arteriography, cardiac catheterization, and interventional procedures are among the many different conditions that can cause embolus formation.[1] Preoperative embolization in the treatment of various tumors may be a cause of such emboli formation and subsequent organ infarction. CRAO as a complication of embolization in the management of JNA has rarely been reported previously.[4,5]

It has been known that inadvertent embolization of the brain or eye during JNA management occurred via dangerous collaterals from the internal maxillary artery to the intracranial/intraorbital contents. Önerci *et al*. presented a patient with JNA who developed CRAO following preoperative embolization. They could not demonstrate any responsible communicating artery and therefore assumed the existence of a branch of the internal maxillary artery supplying the intraorbital contents and the retina in their case. Hence, they recommended more detailed assessment of these possible dangerous collaterals by superselective catheterization of the internal maxillary artery branches with microcatheters.[4] In our case, however, careful retrospective review of the angiograms revealed the presence of a suspicious collateral artery between the external carotid artery and ophthalmic vessels on the left side which had not been noticed before embolization. Thus, it is more probable that the embolus passed mostly via this collateral artery to the left central retinal artery while tumor embolization was being carried out through the left side arteries.

The other pitfall encountered in our case was the delay in the management of CRAO. It was mostly due to the lack of an ophthalmologist in the primary center in which radiologic intervention was performed. To make this presentation concise we will not elaborate on the management of CRAO. However, since the precious time for keeping the retinal cells alive by restoring the retinal artery flow is estimated to be about 90 min,[6] this report underlines the importance and benefits of radiologists’ familiarity with the early diagnosis and immediate therapies of CRAO. Nonetheless, the current recommended treatments may not be more effective than the natural course of the disease.[7,8]

During surgical removal of the tumor, it was noted that the tumor had an extraordinary origin from the midline as opposed to the usual lateral location of the tumor pedicle in the majority of JNA cases. The association of this rare tumor position with the existence of a collateral vessel in our case might have happened by chance and we could not reach any conclusion in this regard.

This case report introduces a patient with CRAO as a rare but an important complication following preoperative embolization of an unusually located JNA tumor. This case also highlights the necessity of careful evaluation of angiograms for detection of any abnormal collateral vessel(s) prior to embolization and the importance of immediate diagnosis and treatment in patients who develop ocular symptoms during or shortly after the interventional procedures.
